# Carbonyl Stress in Red Blood Cells and Hemoglobin

**DOI:** 10.3390/antiox10020253

**Published:** 2021-02-07

**Authors:** Olga V. Kosmachevskaya, Natalia N. Novikova, Alexey F. Topunov

**Affiliations:** 1 Bach Institute of Biochemistry, Research Center of Biotechnology of the Russian Academy of Sciences, 119071 Moscow, Russia; rizobium@yandex.ru; 2 National Research Center “Kurchatov Institute”, 123182 Moscow, Russia; nn-novikova07@yandex.ru

**Keywords:** red blood cells, hemoglobin, reactive carbonyl compounds, reactive oxygen species, methylglyoxal, glycation, glycolytic enzymes

## Abstract

The paper overviews the peculiarities of carbonyl stress in nucleus-free mammal red blood cells (RBCs). Some functional features of RBCs make them exceptionally susceptible to reactive carbonyl compounds (RCC) from both blood plasma and the intracellular environment. In the first case, these compounds arise from the increased concentrations of glucose or ketone bodies in blood plasma, and in the second—from a misbalance in the glycolysis regulation. RBCs are normally exposed to RCC—methylglyoxal (MG), triglycerides—in blood plasma of diabetes patients. MG modifies lipoproteins and membrane proteins of RBCs and endothelial cells both on its own and with reactive oxygen species (ROS). Together, these phenomena may lead to arterial hypertension, atherosclerosis, hemolytic anemia, vascular occlusion, local ischemia, and hypercoagulation phenotype formation. ROS, reactive nitrogen species (RNS), and RCC might also damage hemoglobin (Hb), the most common protein in the RBC cytoplasm. It was Hb with which non-enzymatic glycation was first shown in living systems under physiological conditions. Glycated HbA1c is used as a very reliable and useful diagnostic marker. Studying the impacts of MG, ROS, and RNS on the physiological state of RBCs and Hb is of undisputed importance for basic and applied science.

## 1. Introduction

The concept of “stress” may be considered as both the external impact on an organism and the response to it. At the same time, stress is known to be a universal physiological response to a sufficiently strong influence, characterized by certain symptoms and stages (“general adaptation syndrome” according to Hans Selye) [1]. Further, the term “stress” started to be used in biology and chemistry to describe the effects of certain chemical compounds (or groups of compounds), most often chemically reactive ones, i.e., electrophiles and oxidants.

Currently, metabolism is defined as a network of enzymatic and non-enzymatic (spontaneous) chemical reactions. An integral part of non-enzymatic metabolism is the formation and redox transformations of chemically reactive compounds. An excess of these substances leads to a certain type of metabolic stress being developed: oxidative stress, induced by reactive oxygen species (ROS), nitrosative—by the reactive nitrogen species (RNS), carbonyl—by the reactive carbonyl compounds (or reactive carbonyl species) (RCC), and halogenating—by the reactive halogen species etc.

The main RCC are the linear (non-cyclic) glucose and fructose forms, along with various aldehydes, ketones, ketoaldehydes, and ketoacids, e.g., glyoxal, methylglyoxal (MG), acrolein, malone dialdehyde, 3-deoxyglucosone, lipid oxidation products, etc. Every RCC contains an electrophilic carbon atom of a carbonyl group capable of reacting with nucleophilic nitrogen atoms in amino acids, amino peptides, and guanine bases (non-enzymatic glycation reaction).

Living organisms have evolved various ways to prevent the non-enzymatic glycation. The most effective of these is the glyoxalase system [2,3]. However, when the antiglycemic protection system does not cope with RCC overage, the state of carbonyl stress develops. The concept of carbonyl stress was championed by Baynes in 1991 [4] based on several lines of similarity with the concept of oxidative stress acknowledged in biology already in 1985 [5]. All eukaryotic cells are susceptible to carbonyl stress to a certain extent, including the nucleus-free red blood cells (RBCs) that seem to be one of the most susceptible sensors of the chemically active compounds in many organisms.

In the review, we tried to generalize the data on carbonyl stress in RBC, focusing on metabolism of MG, which, along with glucose, is largely responsible for the negative consequences of diabetic hyperglycemia. We paid special attention to the relationships of carbonyl stress with oxidative and nitrosative stress, and the influences of these processes on Hb.

## 2. Genesis of Carbonyl Stress in Red Blood Cells

**Figure 1:**
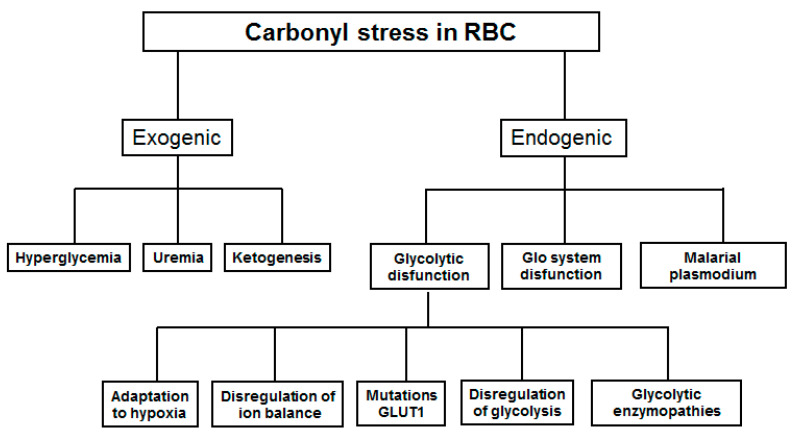
Ways of realization of carbonyl stress in red blood cells. GLUT1—glucose transporter, Glo—glyoxalase system.

There are two major pathways by which carbonyl stress may develop in RBC: exogenic and endogenic (Figure 1). The first pathway is induced by an increased concentration of glucose or ketone bodies in the blood plasma. The second is triggered by misbalance in the glycolysis regulation in RBC itself. Some factors make minor contributions to the development of carbonyl stress here. These include infection with malarial plasmodium, glycolytic enzymopathies, and mutations in the glucose transporter (GLUT1).

The levels of triglycerides, MG, and ROS were shown to increase in the blood plasma of diabetes patients of the first and second types [3,6,7]. Experiments on the isolated RBCs established that the intracellular MG concentration directly depends on the flow of glucose metabolizing in the glycolytic pathway [2]. In the 5–100 mM range of glucose concentration, dose-dependent increases of S-D-lactoglutathione and MG concentrations were registered [2]. MG in plasma can exert several toxic effects: it modifies proteins and lipoproteins, generates ROS, and acts on RBC and endothelial cells. These combined with MG effects facilitate the development of arterial hypertension, atherosclerosis, hemolytic anemia, vascular occlusion, and local ischemia [8,9].

RBC metabolism is defined by a huge role of glucose metabolism since all the necessary energy the cell gets is obtained through the glycolysis reaction and pentose phosphate pathway. The intermediates of the glucose catabolism (glyceraldehyde-3-phosphate (G3P) and dihydroxyacetone phosphate (DHAP)) represent the main sources of MG [10,11].

The glucose for glycolysis is delivered to RBCs in an insulin-independent way using lightweight diffusion via the glucose transporter GLUT1. Thus, the glucose concentration inside RBCs directly depends on its concentration in blood plasma. Therefore, RBCs are among the first cells detecting hyperglycemia. At high concentrations of glucose, oxygen couples with iron ions inside RBCs, leading to the formation of RCC. Although the RBC contains powerful antioxidant and glyoxalase defense systems [3], there is a high probability of carbonyl and oxidative stress development reinforcing each other [12,13]. The main trigger of oxidative and carbonyl stress is that antioxidants and key glycolysis enzymes get damaged by ROS and RCC [14]. For example, the non-enzymatic glycation of superoxide dismutase leads to enzyme inactivation [15,16]; thus, glyceraldehyde-3-phosphate dehydrogenase (GAPDH) becomes incapable of binding the substrate after oxidation or nitrosylation of SH groups in its active center [17]. These spontaneous posttranslational modifications of enzymes result in the accumulation of ROS and triosophosphates—the main source of MG in the cell.

The largest contribution to the pool of endogenic MG is made by the non-enzymatic hydrolysis reaction of the phosphate group of triosophosphates: DHAP and G3P [10,11]. Two enzymes of glycolysis are involved in this metabolism. They are triosophosphate isomerase regulating DHAP and G3P interconversion, and GAPDH oxidizing G3P to phosphoglyceric acid. Once the synthesis and functioning of these enzymes are broken, MG is excessively formed. RBCs with mutant triosophosphate isomerase contain 20–40 times higher amounts of DHAP than in the control variant [18]. The functioning of these enzymes in RBC under carbonyl stress will be described in greater detail in Section 3.

Quite an exotic reason underlying the carbonyl stress developing in RBC can be the malarial *Plasmodium* infection. Malaria was the first disease shown to be caused by a protozoan (1880). Charles Louis Alphonse Laveran received the Nobel prize for this discovery in 1907. Several species of the *Plasmodium* genus (*P. malariae, P. falciparum*, and *P. vivax*) are known to cause this infection. This is a fairly rare case of an intracellular infection, when the protozoan (eukaryote) develops inside such a small cell as a RBC. Plasmodium feeds on the contents of the RBC, primarily Hb, as the main constituent protein of this cell. Both in the drawings made by Laveran as early as in the XIX century from a microscope, and in the modern photographs, one can see how the RBC interior brightens in the course of malaria development.

Vander Jagt et al. have shown that RBCs after infection with the malarial Plasmodium *P. falciparum* secrete ~30 times more D-lactate than as usual [19]. The malaria parasite needs a large amount of glucose to ensure its rapid growth and reproduction inside the RBC [20]. Therefore, the infected RBCs are forced to consume more glucose in order to provide enough resources for the parasitic organism [21]. That is why we attributed this cause of carbonyl stress in RBCs to an endogenic group (Figure 1). Meanwhile, an elevated concentration of glucose in RBCs and a high rate of Plasmodium glycolysis contribute to increased MG formation in the cell and the development of carbonyl stress.

One more exogenic cause of an increased MG level in blood plasma could be intestinal bacteria [22,23]. In this case though, the increase in MG concentration is negligible, so this reason is not indicated on Figure 1.

## 3. Inhibition and Inactivation of the Glycolytic Enzymes

**Figure 2:**
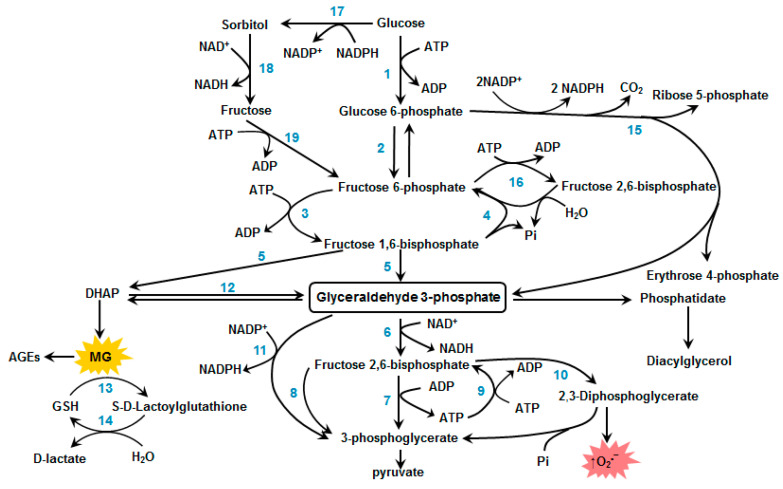
Futile cycles and shunts of glycolytic pathway in red blood cells. Enzymes catalyzing these reaction are designated by numbers: 1—hexokinase, 2—glucose phosphate isomerase, 3—phosphofructokinase, 4—fructose 1,6-bisphosphatase, 5—fructose diphosphate aldolase, 6—glyceraldehyde phosphate dehydrogenase (GAPDH), 7—phosphoglycerate kinase, 8—oxidized GAPD (GAPD-SOH), 9—phosphoglycerate kinase, 10—bisphosphoglycerate mutase, 11—non-phosphorylating glyceraldehyde phosphate dehydrogenase (GAPDH), 12—triose phosphate isomerase, 13—glyoxalase i, 14—glyoxalase ii, 15—glucose 6-phosphate dehydrogenase, 16—fructose 2,6-bisphosphatase, 17—aldose reductase, 18—sorbitol dehydrogenase, 19—hexokinase.

The carbonyl stress affects many reactions and relevant enzymes in RBC. Figure 2 shows the scheme of reactions, futile cycles, and shunts of the glycolytic pathway in these cells. The enzymes catalyzing different reactions of this pathway are labelled by numbers on the scheme. In this section, some of the main glycolytic enzymes, glyceraldehyde-3-phosphate dehydrogenase, triosophosphate isomerase, and enzymes of glyoxalase, system will be addressed in greater nuance.

### 3.1. Glyceraldehyde-3-Phosphate Dehydrogenase

One of the main glycolytic enzymes—glyceraldehyde-3-phosphate dehydrogenase (EC 1.2.1.12)—catalyzes the oxidation of glyceraldehyde-3-phosphate (G3P) to 1,3-diphosphoglycerate. Inhibition or inactivation of this enzyme leads to a significant accumulation of dihydroxyacetone phosphate (DHAP)—a G3P isomer. It is further spontaneously hydrolyzed, leading to MG formation. It can further react with lysine and cysteine residues in GAPDH [24,25], thereby impeding the proper enzyme activity. Normally 0.05–0.1% of triosophosphate intermediates go to MG formation [26].

The oxidative posttranslational modification of cysteine residues in the active center of GAPDH, which are very sensitive, might lead to inhibition of the enzyme. Their mild oxidation with hydrogen peroxide to sulfenic acid (-SOH) dampens GAPDH dehydrogenase activity while stimulating the acylphosphatase one [27].

GAPDH can also be inactivated in cases of deep oxidation of the catalytic cysteines by ROS to sulfinic (-SO_2_) and sulfonic acids (-SO_3_) [28,29], S-nitrosylation by NO donors [30,31,32], and S-glutathionylation [32]. A ROS source in RBC can arise in the reaction of MG with amino acids [13,33], auto-oxidation of glucose [33], and auto-oxidation of oxyHb [34]. GAPDH disfunction is as well brought about by hypochloride-dependent oxidation, when hypochloride triggers the radical’s formation on amino acid residues of the enzyme [35].

MG modifies a glycolysis enzyme, GAPDH, with lysine residues leading to a decrease in the enzyme activity [24,25]. A GAPDH substrate G3P can also act as a glycating agent for the enzyme [36]. However, G3P glycates GAPDH providing the enzyme is inactivated or inhibited and thus unable to effectively convert G3P to 1,3-diphosphoglycerate. As a result, we have an increased concentration of G3P, which is isomerized to dihydroxyacetone phosphate, which MG is further formed from. The causes of GAPDH disfunction are RCC and ROS (e.g., O_2_^•−^), those being formed under hyperglycemia conditions. GAPDH inhibition can disrupt the cell’s energy supply, which is especially critical for RBCs as glycolysis is the sole source of energy for them. Thus, GAPDH modification and inhibition largely contribute to the carbonyl stress in RBCs [36].

GAPDH modification activates the glycolysis futile cycle (Figure 2) and inhibits glycolysis at the level of the triosophosphates conversion [37]. In this case, S-nitrosylation of GAPDH decreases the enzyme affinity to the RBC membrane [31]. GAPDH inhibition may subsequently uncouple oxidation and phosphorylation processes during glycolysis and a decrease in ATP yield. This is especially critical for RBCs, which have no other energy sources than glycolysis.

**Figure 3:**
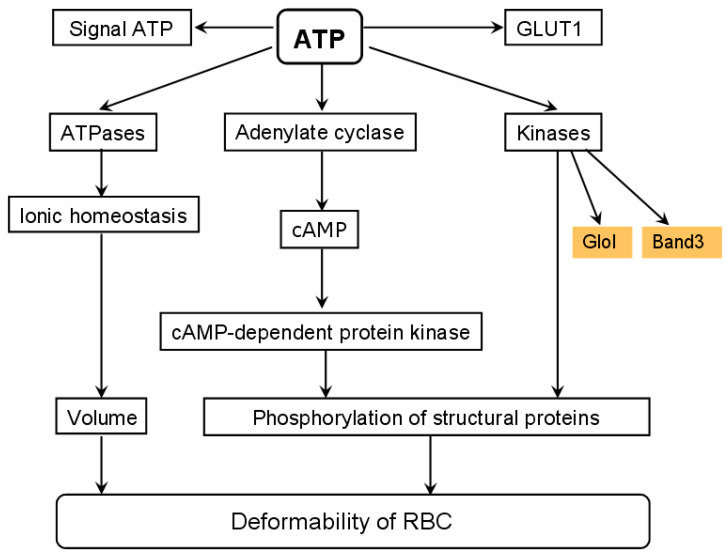
ATP-regulated processes in red blood cells. cAMP—cyclic AMP, Glo1—glyoxalase I, Band3—RBC membrane anion transporter protein (band 3 protein).

The decrease of ATP level leads to disruptions of the RBC shape, volume, and deformability, which all negatively affect their rheological characteristics. For example, the RBC deformability of patients with type 2 diabetes is significantly lower than that of healthy people [38]. Figure 3 shows the scheme of ATP-dependent processes in RBCs.

MG was reported [39] to decrease not only the energy production, but also antioxidant protection, which contributes to the eryptosis (apoptosis of RBCs [40]) of the circulating cells. In general, there is a clear correlation between carbonyl and oxidative stress [12,41,42]. On the one hand, in the reaction of amino acids and proteins with MG, free-radical intermediates (MG anion radical and cation radical of a Schiff base) are formed along with ROS and RNS [13,34,43,44]. On the other hand, ROS and RNS themselves can contribute to formation of new AGEs [45,46]. This situation was most aptly called “a vicious circle.”

In vitro experiments on RBC incubation with high glucose concentrations simulating hyperglycemia showed significant increases in the levels of triosophosphate intermediates (G-3-P and DHAP) and MG, proportional to the glucose concentration [2,47,48].

In vivo experiments of type 1 and type 2 diabetes patients revealed an inverse correlation between MG production and GAPDH activity. A 79% decrease in GAPDH activity in the culture of human RBCs led to a six-fold increase in MG concentration [49]. Herewith, MG in high concentrations inhibited glycolytic enzymes: GAPDH, phosphofructokinase, fructose-1,6-diphosphatase, aldolase, and 3-phosphoglycerate mutase [37].

### 3.2. Triosophosphate Isomerase

The enzyme triosophosphate isomerase (TPI) (EC 5.3.1.1) catalyzes the DHAP conversion to G3P (Figure 3). The lack of TPI or decrease in its activity leads to accumulation of DHAP, which spontaneously decomposes, with MG being formed as an outcome [50]. Importantly, DHAP and the already formed MG act as glycating agents.

It was shown that TPI activity gradually decreases during the catalytic function unfolding [51]. This accounts for the spontaneous deamidation of Asn15 and Asn71 residues, and their conversion to aspartic or isoaspartic acid [51,52]. The modified enzyme dissociates to monomers, which then undergo proteolytic degradation [53]. Hipkiss suggested that continuous and excessive glycolysis promotes TPI deamidation [54,55]. This situation is typical for diabetic hyperglycemia and a diet with a high glycemic index.

Since mature RBC are not able to synthesize new TPI as they do have neither nuclei nor ribosomes, MG gets inevitably stored there with aging. A high activity of TPI from RBCs, being several times higher than the activity of other glycolytic enzymes, might be seen as an evolutionarily developed adaptation compensating for the decrease in the TPI activity during a RBC’s life [55].

TPI dysfunction can also result from the nitration of two tyrosine residues in the active center, induced by amyloid-β-peptide aggregates [56]. Phenylpyrrole fungicides, actively used to prevent plant infection with various pathogens, also demonstrated an inhibitory effect on TPI [57].

### 3.3. Glyoxalase System

The glyoxalase system is a highly conserved enzymatic system that evolved to maintain MG concentration in cells at a low (about nanomolar) non-toxic level [58]. In RBCs, the glyoxalase pathway throughput is by two orders higher than that for glycolysis [59].

The glyoxalase system includes two enzymes: glyoxalase I (GloI, S-D-lactoylglutathione lyase, EC 4.4.1.5), glyoxalase II (GloII, hydroxyacylglutathione hydrolase, EC 3.1.2.6) and reduced glutathione (GSH) [2,3,60,61,62]. The system catalyzes the reaction continuity of α-oxoaldehydes conversion to the corresponding α-hydroxy acids using GSH as the cofactor. Thiohemiacetal is spontaneously formed in the reaction of α-oxoaldehydes with GSH. GloI isomerizes it to S-D-lactoylglutathione, which is hydrolyzed to α-hydroxy acid (to D-lactate in the case of MG) and reduced glutathione under the action of GloII.

GloI and GloII were isolated from human RBCs and characterized. GloI is a homodimeric protein containing Zn^2+^ with 46 kDa molecular weight of a dimer [63]; GloII is 29.2 kDa monomer [64]. The catalytic parameters of GloI and GloII from human RBC were evaluated as well: for GloI K_m_ = 192 ± 8 µm and K_cat_ = 10.9 ± 0.2 × 10^4^ min^−1^ [63]; for GloII K_m_ = 146 ± 9 µm and K_cat_ = 727 ± 16 s^−1^ [64]. According to [65] native RBC GloI has K_m_ = 0.7 ± 0.1 mm and V_max_ = 0.7 ± 0.04 µm/min. It is GloI that limits the speed of the glyoxalase system.

Glyoxalases’ disruption leads to the MG accumulation in the cell. Glyoxalases are inactivated or made dysfunctional in the process of oxidative posttranslational modifications. GloI activity can also be reduced under nitrosylation by physiological NO donor S-nitrosoglutathione [66,67]. Another modification of the enzyme leading to its inactivation is glutathionylation by Cys139 [65], but it is less possible. The ability of GloI to perform reverse oxidative modification implies that enzyme activity depends on the red-ox state of the cell. Since GloI activity is proportional to GSH concentration, another reason for high intracellular MG concentration may be the depletion of the pool of reduced glutathione due to oxidative stress [46].

In this regard, the recent cycle of studies on ROS formation under the impacts of various factors is also worth mentioning. Among these factors: nicotine in smokers [68], wood dust [69], crystalline silica [70], and ionizing radiation from cancer treatment [71]. ROS formed lead to GloI inhibition, enhancement of the MG-induced formation of AGEs—potent pro-apoptotic agents, and finally, to apoptosis of different cells in the organism.

In addition to some oxidative modifications, the GloI stability and activity can be regulated by acetylation and phosphorylation processes [65]. Diabetic hyperglycemia or a high-carb diet also promotes intense and persistent glycolysis, which increases the MG concentrations [55]. The diabetic patients exhibited several signs of oxidative stress: oxidation of lipids and SH- groups of erythrocyte membranes, and a decrease of GSH level [72,73]. The increased MG concentration observed at diabetic hyperglycemia may be associated both with increased MG production during glycolysis [39], and a decrease in GloI activity, e.g., as a result of the oxidation by ROS [69,74,75]. Therefore, even at normal glucose concentrations, patients with type 1 diabetes had a 25-fold increase of MG level, and with type 2 diabetes—1a 5-fold increase [76].

The decreased GloI activity in RBC and MG accumulation was observed in Alzheimer’s patients [77]. Moreover, it was accompanied with the accumulation of the lipid peroxidation products and a decrease in the 20S proteasome activity. This means that there is a direct correlation between a reduced glyoxalase activity and oxidative stress itself. The amplification of dicarbonyl stress by oxidative one has been discussed in some papers [45,46,78]. High MG levels were found in RBCs of the end-stage renal failure patients undergoing hemodialysis. It may be associated with impaired glycolysis in the cell [79].

Since glyoxalase activity decreases with age [80], the old RBCs are the most likely source of MG, as MG can induce RBC lysis. Consequently, RBCs become a systemic source of MG and glycated proteins [39]—in particular, glycated alpha-synuclein [55,81,82]. In the course of a RBC’s life, the activity of the glyoxalase system changes [75], it is maximal in mature cells, and during RBC aging the activity decreases. The decrease in the GloI activity in RBC in this case seems to be connected with its oxidative modification [80,83].

Hereby, the MG accumulation in RBC can result from high glucose concentrations, oxidative stress, and cell aging.

## 4. Effects of Methylglyoxal on Red Blood Cells

### 4.1. Methylglyoxal—Highly Reactive Dicarbonyl Metabolite

Special consideration should be given to the above mentioned α,β-dicarbonyl compound—methylglyoxal (2-oxopropanal, pyruvate aldehyde, pyruvaldehyde). Its glycating activity is 20 thousand times higher than that of glucose or fructose [11]. The toxic effect of MG arises from its ability to react with amino and SH groups of protein amino acid residues, forming covalently bound advanced glycation end products (AGEs): N^ε^(carboxyethyl)lysine, N^ε^(carboxymethyl)lysine), imidazolones, argpyrimidines, MG-derived lysine dimers, and thiohemiacetals [11,84]. These modifications are irreversible, and induce changes in structure, charge, conformation, and ultimately, lead to protein dysfunction [85].

Rabbani and Thornley proposed the term “dicarbonyl stress,” thereby emphasizing that α-ketoaldehydes (mainly MG) are the main physiological precursors of AGEs and are responsible for many pathological effects [11,84].

MG concentration in RBC increases during diabetic hyperglycemia [2,3,86] and aging [87]. Moreover, this metabolite can be formed both in RBC themselves, and come from the blood plasma [88]. Depending on the concentration, MG may play either a toxic or a regulatory role [89,90,91,92]. High doses of MG severely damage the biomolecules and excessively activate the intracellular signaling pathways, while the ones can function as signaling molecules, modulating different aspects of the cell behavior. In the eukaryotic cells, MG has been shown to interact with the signaling pathways regulating proliferation, apoptosis, growth factor production, and other physiological functions [91].

At the molecular level, the signaling and regulatory MG actions are often realized through changes in the so-called cysteine proteome—a complex of protein cysteine residues, especially the reactive ones. The targets of MG in RBC are thiol groups of hemoglobin (Hb) [93], glyceraldehyde-phosphate dehydrogenase (GAPDH) [94], and RBC membrane anion transporter protein—the so-called band 3 protein (Band 3) [95]. These proteins determine the level of cellular energetics, and RBC ability for gas transport largely depends on their functioning. In addition, Band 3 is the main site of Hb binding to the RBC membrane [96,97,98,99].

### 4.2. Pathological Effects Caused by MG Action on RBC

Many glycolytic enzymopathies were clinically revealed to accompany chronic hemolytic anemia [39]. Reactive aldehydes and ROS induced by them play a significant role in triggering their pathological consequences. In vitro experiments have shown that RBC incubation with MG leads to membrane destabilization, accompanied by cell lysis [7].

As mentioned above, there is a clear link between RCC and ROS formation. The RCC are now considered to be the markers of oxidative stress and RBC aging [100]. The chronic hyperglycemia and the associated metabolic disorders are known to affect the membrane, which is important for RBC’s functional activity. RCC and ROS are mostly dangerous for the RBC membrane structures, the lipid bilayer, receptors, ion channels, transporters, and membrane-bound enzymes, as they are poorly protected by superoxide dismutase [101]. High RCC concentrations in blood plasma positively correlate with the amounts of carbonylated RBC membrane proteins [100].

It was shown that in rats with alloxazan diabetes, the number of irreversibly altered RBC increased with the prolongation of hyperglycemia. One of these alterations is to RBC form—their conversion to the so-called spherocytes. At the third-fourth week, the portion of spherocytes was 55% compared to 20% in intact animals [102]. The injury of RBC membrane components affects the elastic-mechanical properties of the membrane, which determines the cell functionality [103,104], and it is a risk factor for the development of the arterial hypertension [105]. One of the diabetes implications may be various microangiopathies emerging from the destruction of RBCs and endothelial cells with RCC. The changes in the elastic-mechanical properties of RBCs among diabetes patients were repeatedly reported. Such RBCs have increased the membrane viscosity and stiffness [104]. It was demonstrated in several model systems in vitro that the observed impairments of RBC properties arise from the action of RCC on membrane components [102]. The membrane damage negatively affects its mechanical properties and integrity, increasing the probability of hemolysis and Hb release into the bloodstream. Both reactive blood plasma compounds (ROS, RNS, and RCC) and Hb itself can affect a RBC’s membrane.

**Figure 4:**
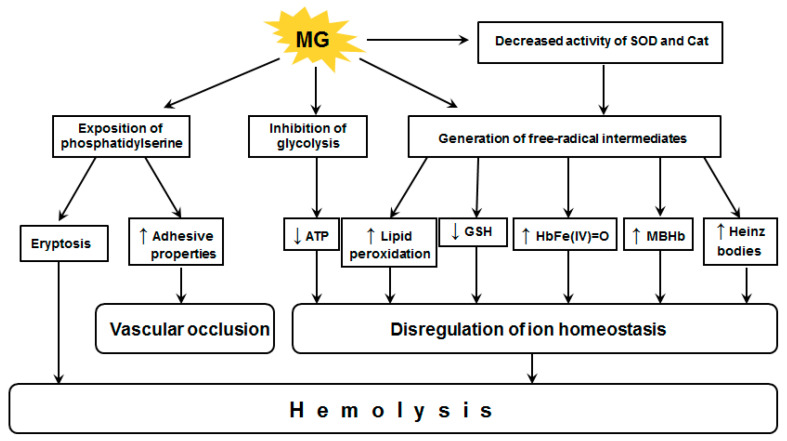
Pathological effects caused by MG action on red blood cells. Cat—catalase, GSH—reduced glutathione, MBHb—membrane-bound Hb; SOD—superoxide dismutase. The arrows ↑ and ↓ in frames indicate an increase and decrease in the indicators, respectively.

The above-described pathological effects caused by MG action on RBCs are generalized and systematized on Figure 4.

In addition to affecting RBC, MG engages in non-enzymatic glycation reactions with blood plasma proteins and factors, disrupting or inhibiting their functions. MG inhibits the activity of anticoagulation factor antithrombin III, forming covalent bonds with Arg393 [106]. This brings about a fibrinolytic activity phenotype in a diabetic hyperglycemia. An elevated MG level in plasma induces an inflammatory response via enhanced expression of cell adhesion molecules, promoting the leukocyte interaction with the endothelium. MG has been shown to activate SGK1 (serum and glucocorticoid-inducible kinase 1) via ROS formation. Meanwhile, SGK1 regulates many endothelial ion channels, including the Na+/H+ ion exchanger [107].

## 5. Carbonyl Stress and Hemoglobin

### 5.1. Glycated Hemoglobin

Structural Hb changes can be both genetically determined and triggered by several spontaneous reactions, e.g., non-enzymatic glycation (interaction with MG) and glycosylation (interaction with glucose). In the literature these terms are often confused and both types of modification are referred to as glycation.

The reaction of RCC with nucleophilic nitrogen atoms in amino acids, peptides, and proteins leads to the appearance of N-substituted glycosamines (so-called Shiff bases). The latter undergo the Amadori rearrangement and form ketamines [84] subject to multiple dehydration and precursors of advanced glycation end products (AGEs)—chemical compounds including pyrrole, pyrazine, imidazole, and furan derivatives. The set of reactions resulting in AGEs formation was first proposed by the French biochemist and physician Louis Camille Maillard in 1912 [108], and then described in detail by John Hodge in 1953 [109].

Although Maillard’s discovery is more than a century old (1912) [108], this reaction was the subject of food chemistry for most of that time. The prospects of this non-enzymatic reaction in biological systems were revealed with the discovery of the glycated Hb. It was the first protein the non-enzymatic glycation of which was deliberately assessed in the living systems under physiological conditions. As early as in 1958 several fractions were detected during the chromatographic separation of human Hb [110,111]. Only 10 years later, the minor fraction of “abnormal fast-moving hemoglobin band” was identified as the Hb adduct with glucose and indicated as HbA1c [112]. The term HbA1c shows the location of this Hb fraction when separating hemoglobin using cation exchange chromatography. The mass spectrometry technique resulted in isolating more Hb subfractions [113]. The proposed mechanism of Hb glycation is as follows. Firstly, the glucose binds to N-terminal valine of β-HbA1 subunits, and then a subsequent rearrangement into 1-deoxy-1-N-valyl-fructose leads to the formation of glycated Hb fraction.

Starting with 1968, studies into the glycated Hb accelerated dramatically. That year Samuel Rahbar reported that the proportion of the glycated Hb increased in RBCs in patients with diabetes [30,114], and in 1969 he published a detailed study of HbA1c in patients with diabetes [115]. In 1975 several reactions leading to HbA1c formation were described [116]. A year later the Cherami group proposed the glycated Hb as a marker of the glycemia level in diabetic patients [117].

Hb is a long-living protein, staying in RBCs for about 120 ± 20 days. This time is sufficient enough for glucose to react with the terminal valine amino group and to form a stable complex. The amount of HbA1c reflects the blood glucose level for a 4–6 weeks period [114,118], while a standard blood glucose test shows its level at the time of measurement, which may not be related to hyperglycemia conditions.

The discovery of HbA1c gave rise to a new research area related to studying the Maillard reaction in biological systems. The presence of glycated proteins and other products of non-enzymatic glycation in biological fluids and tissues proves that these processes take place in living organisms. It was shown both under normal conditions and in various pathologies. Thus, under hyperglycemic conditions, the Amadori products associated with amino groups of α- and β-Hb subunits appear [119].

In diabetes patients, a negative correlation between the level of HbA1c and the activity of Na+/K+-ATPase in RBC has been established. This correlation is most likely associated with the glycation of the membrane proteins in conditions of high sugar content [120].

The level of glycated proteins also increases with aging of the organism [121,122,123,124].

### 5.2. Hemoglobin in the Development of Carbonyl Stress Consequences

The extent to which RBCs can be damaged largely depends on the processes going on with Hb. Hb is a “long-living” protein that extensively accumulates various posttranslational modifications, including non-enzymatic ones. There are several known spontaneous biochemical reactions with amino acids in proteins, i.e., not catalyzed enzymatically. They are: (1) oxidation of cysteine, tyrosine, and tryptophan residues; (2) nitrosylation of cysteine; (3) nitration of tyrosine, tryptophan, and methionine; (4) chlorination of lysine, methionine, glycine, and arginine; (5) formation of labile (Schiff bases, Amadori products) and stable amino acid adducts (AGEs).

The following posttranslational modifications of Hb were also identified: glycation under diabetic hyperglycemia, acylation under alcoholism, chronic renal failure, formation of a cyanate adduct under uremia, etc. Thus, specific forms of the modified Hb can be diagnostic of several metabolic disorders. The complex data on different Hb forms, including the modified forms, are proposed to be used in a computer expert system to diagnose anemia and hemoglobinopathies and other diseases manifesting these symptoms [125]. This system, along with the artificial neural networks [126], was recognized as the most promising direction for hematological diagnostics [127].

Hb in RBC can exist in both soluble and membrane-bound forms. The ratio between them correlates with the Hb state and the conditions of RBC membrane. The reversible binding of Hb to the membrane is an adaptive process and can adjust properties of the membrane and carbohydrate metabolism if the conditions are changing, e.g., partial oxygen pressure (pO_2_) [128,129]. In case of the oxidants’ action, irreversible covalent Hb binding to the membrane components may take place, which destabilizes the membrane and leads to Hb release into blood plasma. The binding of Hb to the membrane is also affected by the MG and ROS-caused Hb modifications under carbonyl and oxidative stress [99]. The structural disorders in Hb are accompanied by the molecule destabilization, the loss of a cooperative effect, and a decrease in the resistance to oxidants. As a result, Hb accumulates in the perimembranous region, the damaged Hb forms aggregates (Heinz bodies), heme is released, and binding to the membrane takes place. It was found that HbA1c is bound to RBC membrane significantly more weakly than normal Hb (HbA0) [130], and has a substantially higher affinity to oxygen [131]. The oxidative Hb modifications may result in reduced hemolytic stability and deformability of RBC, and their hindered movement through narrow capillaries. It was shown that the pathologically altered RBCs have a tendency towards aggregation, apoptosis, and hemolysis [132,133]. RBC hemolysis entailing the release of Hb into a vessel is an extremely undesirable phenomenon leading to a number of physiological disorders in the cardiovascular system. However, it should be noted that the Hb transition to the membrane-bound state contributes to the realization of several specific signal-regulatory functions [99].

There are three possible mechanisms of toxic Hb action. The first is the development of vasoconstriction resulting from NO oxidation to nitrate during reactions with oxyHb (NO-dioxygenase reaction). The second involves the formation of active radical products—superoxide-anion radical, peroxynitrite, and ferryl- and oxoferrylHb—inducing the oxidation of the low-density lipoproteins in plasma. The third mechanism is based on the reactions of a free heme, inducing the ROS formation and emergence of inflammatory mediators via activating NF-kB transcription factor in endothelial cells [134] and macrophages and neutrophils [135,136]. All these phenomena lead to impairments in blood rheological properties and to vascular occlusion [136,137,138]. Therefore, to successfully search for medications reducing the degree of hemolysis under carbonyl and oxidative stress conditions, the mechanisms of RBC stabilization are to be investigated down to the last detail.

### 5.3. HbA1 and MG—the Hyperglycemic Biomarkers

The toxic effects of elevated glucose result in microvascular (retinopathy, neuropathy, and nephropathy) and macrovascular complications, ultimately leading to the development of heart failure. To reduce the risk of microvascular and macrovascular consequences such as diabetes, optimal glycemic control is required.

In 2009 a committee of the American Diabetes Association recommended HbA1c as an official indicator for diagnosing diabetes. The glycated Hb proved to be a highly reliable and useful diagnostic marker, since every 1% increase in HbA1c level correlates with a 15–18% increase in the risk of developing cardiovascular diseases [139]. The level of glycated Hb was called the “golden standard” for hyperglycemia and blood glucose testing [140].

However, the use of HbA1c as an ideal glycemic marker is currently disputed [141]. The HbA1c test is not always applicable. Particularly, there are restrictions for aplastic anemia patients with type 2 diabetes [142]. In addition, HbA1c does not reflect the degree of the glycemic and glycooxidant damage under diabetes [143]. Better suited for this purpose could be specific plasma AGE biomarkers, which are products of MG-caused glycation: Nε(carboxyethyl)lysine, Nε(carboxymethyl)lysine, and MG-derived hydroimidazolones [140,144,145,146]. For instance, the level of these AGEs predicts the rapid progression of the diabetic nephropathy [147]. It is supposed that detecting the autofluorescence of the skin AGEs allows one to assess the risks of diabetic sequela [148,149]. Note that the fasting plasma glucose level was significantly positively correlated with serum MG.

It is known that during glycation (interaction with MG), the most effectively modified amino acid residue is the lysine located near the histidine imidazole ring, and an imidazole group was found to catalyze the the Amadori rearrangement. This mechanism is used for the glycation of free amino groups in Hb, and in albumin and some other proteins [150,151,152].

Recently, the role of MG as a biomarker for early detection and monitoring of long-term metabolic complications has been actively discussed [153]. The MG plasma level is a risk factor predicting the progress of macro- and microvascular disturbances of type 2 diabetic patients, and the intima-media thickening, vessels rigidity, and systolic blood pressure [154]. It proves the clinical significance of MG as a biomarker for diabetic macroangiopathy. Tests for MG in blood plasma are being developed, which will allow one to forecast vascular impairment at the early stages of the disease [153].

There are several methods allowing for direct estimation of MG concentration in blood plasma: high-performance liquid chromatography, electrochemical biosensors, electrospray ionization-liquid chromatography-mass spectrometry, enzyme immunoassay, and capillary electrophoresis [154,155,156]. Some of them are also applicable for intracellular MG detection. In [155], a selective fluorescent sensor (methyldiaminobenzene-BODIPY) for MG identification in the cells was proposed. Additionally, recently a fast, simple, and cost-effective method for MG quantitative detection in blood has been reported, based on the far-infrared spectral analysis of the product of MG reaction with o-phenylenediamine [157].

For a more accurate assessment of the risk of cardiovascular diseases at the early diabetes stages, the combined use of several parameters was suggested: fasting blood glucose, blood pressure, the amount of glutathione in RBCs, and total cholesterol [158]. Gycated albumin, fuctosamine, and 1,5-anhydroglucitol were also proposed as alternative glycemic markers [141]. These biomarkers are not yet used in the clinic practice due to the lack of standardization.

## 6. Relationship of Carbonyl Stress with Oxidative and Nitrosative Stresses

When studying the effect of RCC on the biological system, it is crucial to take into account their interactions with other reactive substances, primarily with ROS and RNS. For example, excessive MG accumulation in cells can lead to the formation of ROS and AGEs, inactivating the cell’s antioxidant systems [159]. Note that MG can participate in a redox relationship with nitrogen oxide (NO) formed in the nitrite reductase reaction catalyzed by deoxyHb [160]. MG can also react with superoxide anion radical (O_2_^•−^) released during Hb auto-oxidation [34]. These reactive compounds can interact with each other, and with SH groups, heme, and non-heme iron, which leads to shifts in the thiol-disulfide equilibrium and in Hb state [41,161]. Free iron ions and the ones included in heme and non-heme complexes play an important role in the coupling of glycation and ROS formation processes [162].

The worse the hypoxia and the larger the deoxyHb portion, the higher the O_2_^•−^ and NO concentrations in RBCs [163]. Moreover, under the reduced oxygen concentration, the intensity of glycolysis increases, which is accompanied by a rise in MG production [164]. Nitric oxide interacts with heme iron and cysteine residues, and Hb has eight binding sites to the MG molecule [93]. Both MG and NO can act as allosteric effectors increasing the Hb affinity to oxygen [165,166], and thereby shift the Hb equilibrium towards the R-conformation.

The signal functions of superoxide and nitric oxide in RBC have been addressed in several comprehensive studies [167]. However, there is still no clear evidence of a signal-regulatory action of MG in these cells. We suppose that it can be manifested by interfering with non-enzymatic reactions caused by O_2_^•−^ and NO.

We have previously investigated the effect of the NO donors on the non-enzymatic Hb modification with MG [41,161]. It was found that *S*-nitrosoglutathione (GSNO) increased the formation of radical intermediates of the lysine reaction with MG, which lead to Hb reduction and nitrosylation. On the other hand, GSNO inhibits Hb modification by MG and the protein crosslinking.

**Figure 5:**
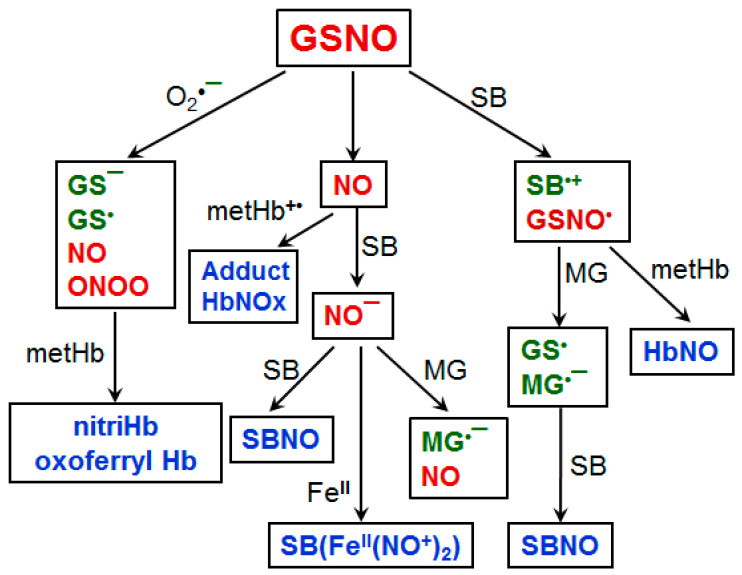
A set of products and free-radical intermediates formed in the reaction of S-nitrosoglutathione with MG in the presence of Hb. R-SNO—nitrosothiols (nitrosoglutathione, nitrosocysteine); SB—Schiff base; SBNO—nitroso adduct of Schiff base; SB(Fe^II^(NO^+^)_2_)—dinitrosyl iron complexes associated with Schiff bases; nitriHb—Hb nitrated at the vinyl group of the porphyrin ring; metHb—oxidized Hb, metHb^•+^—the Hb-bound Schiff base radical cation, oxoferrylHb—Hb-Fe^IV^ = O.

At the same time, GSNO does not provide any cytoprotective action for the RBCs pre-treated with MG. GSNO in some cases is a source of RNS, causing an irreversible modification of the porphyrin and the formation of nitrimetHb. The obtained results are summarized in Figure 5. It reveals the main pathways in the formation of products and free-radical intermediates occurring in the Hb−GSNO−MG system. This diagram illustrates the transformations network that accompanies the exchange of the NO metabolites, RCC, and Hb in normal and pathological conditions. The presence of oxygen and/or NO in the system determines the degree of covalent Hb modifications.

Studying the impacts of MG and NO, being critical bioregulators of RBCs, on their physiological state, is of undisputed importance for basic and applied science. These substances are of particular interest because they can be both present in blood plasma and be formed in the RBCs themselves. Depending on the concentration, they can display either toxic or regulatory effects. The toxic effects are mostly elucidated; but the signaling and regulatory actions are still largely unknown, which is especially true for MG [91]. Last but by no means least, studying the role of these compounds, in implementing the program of adaptation to various pathologies, seems to be a promising direction for future research.

A positive correlation was found between the HbA1c level and the severity of oxidative stress in RBC of diabetes patients [168,169]. In their blood, as compared to the control group, the concentration of malondialdehyde—a product of lipid peroxidation—was increased, and the content of GSH—a substrate of glutathione peroxidase and glutathione reductase, was reduced [168,169,170]. In diabetic patients the activity of glucose-6-phosphate dehydrogenase was reduced. This activity is involved in GSH redox cycle along with glutathione peroxidase and glutathione reductase [169]. A decrease in the activity of these enzymes leads to oxidative stress and the accumulation of the oxidized denatured Hb forms, which trigger the eryptosis process (so-called quasi-apoptosis of RBC) [143]. It promotes the development of hemolytic anemia [171], the prevalence of which in patients with diabetes mellitus is estimated at 22% [170] or 18% [172].

Oxidative and carbonyl stress promote the oxidation of intracellular GSH, which is consumed in the reactions with antioxidant enzymes and glyoxalase system. Thus, in the RBCs of patients with type 2 diabetes, the GSH pathway was more susceptible to oxidation, if compare to the control group [173]. In RBCs of diabetic patients, depletion of GSH levels was observed due both forming conjugates with MG, and interaction with lipid peroxidation products (e.g., 4-hydroxy-2,3-nonenal) [174]. At the same time, under the conditions of hyperglycemia, the synthesis of GSH in the RBCs was not disturbed [175]. RBC morphology and functional state depends on the GSH pool [176]. GSH is also involved in maintaining vascular tone and carbohydrate metabolism. Infusion of glutathione decreases blood pressure and potentiates insulin secretion in patients with insulin resistance and impaired glucose tolerance [177].

Hyperglycemia and the associated oxidative stress affect the RBC’s biochemistry and morphology, which interfere with function and life duration. In RBCs both incubated with glucose in vitro and isolated from the blood of diabetic patients, a decrease in deformability, increased susceptibility to hemolysis, increased ROS production, and accumulation of oxidative damage were observed [178,179]. In such glycated RBCs, as a result of eryptosis, phosphatidylserine was exposed on the cell surface, which leads to increased phagocytosis by endothelial cells [179]. Biochemical shifts in RBC are accompanied by morphological changes. Treating RBCs with MG in vitro leads to the transformation of normal biconcave cells into echinocytic ones [180]. In RBCs obtained from the blood of diabetic patients, hypochromia and anisopoikilocytosis (the spread of RBCs by size and shape) were observed [181].

Altered structural and functional RBC states affect the hematological parameters. Thus, in the diabetic patients the values of the RBC parameters were reduced: the average Hb concentration, the average cell volume, and hematocrit [181,182]. At the same time, the width of RBC distribution of diabetic patients was significantly increased. Changes in hematological parameters facilitate vascular damage, which leads to long-term macro- and microvascular impairment [183].

## 7. Pharmacological Interventions and Future Perspectives

There are several possible strategies to deal with carbonyl stress in RBCs. They include using glyoxalase activators and anti-glycation agents (ACS traps), suppressing glycolysis, and switching glucose metabolism from glycolysis to the pentose phosphate pathway.

Currently, low molecular weight compounds enhancing the activity or GloI expression are of particular interest [74,184]. A synthetic substance (candesartan) and substances of the natural origin (resveratrol and fisetin) can serve as these enhancers. Unlike GloI activators, MG traps interact directly with the RCC, thereby reducing its concentration. These are pyridoxamine, aminoguanidine, alagebrium, and benfotiamine [185].

A promising pharmacological agent slowing down pathological processes under hyperglycemia is the natural dipeptide carnosine (β-alanyl-L-histidine). The concentration of carnosine in RBCs is ~10 times higher than in blood serum [82]. Carnosine has been shown to prevent the formation of MG-induced AGEs or even reverse AGEs previously formed [186]. Carnosine also promotes the proteolysis of aberrant proteins and exhibits viable antioxidant properties [187]. There is a point of view that carnosine affects glycolysis by reducing ATP synthesis [187,188]. The reason behind it can be the ability of carnosine to activate fructose 1,6-bisphospatase, which converts fructose-1,6-bisphosphate to fructose-6-phosphate [189]. Thereby, carnosine starts the futile cycle, while reducing both energy production and MG formation (Figure 3). Various studies have shown a positive effect of carnosine on the morphology and deformability of RBCs [190,191,192,193]. It can be partly explained by antioxidant properties of carnosine, which can protect RBCs against the ROS action, which cause peroxidation of membrane lipids. Such peroxidation can decrease RBC deformability and impair microcirculation. Moreover, the oxidative stress in RBCs often stems from diabetic hyperglycemia [194]. Carnosine has been shown to reduce lipid peroxidation, and to normalize RBC deformability in rats with streptozotocin-induced diabetes [192]. Carnosine also prevents alterations in rheological characteristics of RBCs incubated in glucose solutions [195].

In this regard, we would like to note the following. We have previously shown, that physiological metabolites of NO—dinitrosyl iron complexes (DNICs) can protect both: Hb against peroxide-caused oxidative stress [196,197] and RBCs against hemolysis induced with hypochlorous acid [198]. Taking into account that we have obtained DNICs containing carnosine as a ligand [199], it is possible to suggest that carnosine DNICs can protect RBCs under carbonyl stress, by combining the protective properties of both components.

Another class of substances counteracting the glyco-oxidative stress is dietary polyphenols [200,201,202,203]. In [197], it was shown on the culture of rat hepatocytes, that ferulic acid and related polyphenols (caffeic acid, ρ-coumaric acid, methylferulate, ethylferulate, and ferulaldehyde) decreased cytotoxicity and oxidative stress caused by glyoxal and MG. Under the impact of these compounds, the formation of ROS and carbonylated proteins was diminished, and mitochondrial membrane potential was improved. We can assume that dietary polyphenols will be effective in fighting the carbonyl stress in RBCs. Zinc oxide nanoparticles (ZnO-NP) synthesized from an aqueous extract of *Morus indica* leaves were also reported as promising pharmacological agents with therapeutic potential at diabetic sequela [180].

One of strategies to circumvent carbonyl stress is to decrease the flow of glucose metabolized via glycolysis. For RBC, it can be achieved through several strategies: either by following a low-carb diet [82], or by switching glucose metabolism from glycolysis to the pentose phosphate pathway. It was shown [47], that thiamine intake stimulates the anaerobic pentose phosphate pathway by increasing the activity of transketolase. It increases the glyceraldehyde-3-phosphate metabolism, and consequently decreases the MG production.

All these strategies, sole or combined, can be used to prevent potential complications in case of diabetes, obesity, cardiovascular diseases, and neurodegenerative disorders.

## 8. Conclusions

Carbonyl stress affects all tissues of the organism, including blood and RBCs. Circulating RBCs are exposed to both RCC of blood plasma (exogenous carbonyl stress) and intracellular RCC (endogenous carbonyl stress). A novel term “glycated RBC” has been recently coined to refer to those cells, which are exposed to high glucose or MG concentrations.

Since mature RBCs lack a biosynthetic apparatus, their proteins function as AGEs accumulators. One of the main target proteins for RCC is Hb, which forms adducts with glucose (modification by N-terminal valine), and with MG (modification by lysine and cysteine residues). The glycation modification leads to a change in the structure and function of Hb, which affects its oxygen-binding property and signaling functions, realized through Hb binding to the membrane protein Band3. Both Band3 and GAPDH can be glycated, and they are both responsible for the level of cellular energy, and hence the physiological state of RBCs.

Carbonyl stress often increases the oxidative one. O_2_^•−^ is formed during the oxidation of free radicals of endiols (intermediates of the Maillard reaction). ROS are also produced during the co-oxidation of sugars and lipids. In the reaction of MG with amino acids, along with MG anion radical, a dialkylamine cation radical is formed. The production of these free radicals is stimulated by physiological NO derivatives, such as S-nitrosothiols. The formation of free radical products leads to the oxidative modification of biomolecules. In particular, the modified Hb forms such as nitriHb, oxoferrylHb and HbNOx adducts are formed.

Finally, modifying the key RBC proteins via glycation and oxidation processes gives rise to the cells with altered morpho-functional characteristics, predisposed to eryptosis. The intensification of eryptosis occurs under diabetic hyperglycemia. Due to this process, on the one hand, the defective RBCs are eliminated, and hemolysis is prevented, but on the other hand, intensive eryptosis can lead to anemia and impaired microcirculation. In addition, glycated RBCs become a source of toxic molecules: O_2_^•−^, H_2_O_2_, MG and glycation alpha-synuclein [204]. In this case, the export of O_2_ and ATP and NO signaling molecules is disrupted. All this leads to the development of vascular pathologies. Therefore, it is crucial to study those substances that can activate or inhibit eryptosis.

To normalize the RBCs’ physiological function, the effective glycemic control is required. A new promising glycemic biomarker is MG. Blood plasma MG, in contrast to the generally accepted glycemic marker HbA1c, makes it possible to predict cardiovascular disturbance at the early diabetes stages. A routine examination of hematological parameters, including an assessment of abnormalities in RBC parameters, will also help to reduce the negative effects of diabetes on the cardiovascular system. Another important field of research in this area is the active search for substances—potential pharmacological agents with both anti-glycating and antioxidant properties, which would minimally affect the structure of biomolecules.
